# Prediction of prostate cancer aggressiveness using magnetic resonance imaging radiomics: a dual-center study

**DOI:** 10.1007/s12672-024-00980-8

**Published:** 2024-04-16

**Authors:** Nini Pan, Liuyan Shi, Diliang He, Jianxin Zhao, Lianqiu Xiong, Lili Ma, Jing Li, Kai Ai, Lianping Zhao, Gang Huang

**Affiliations:** 1 The First Clinical Medical College of Gansu University of Chinese Medicine, Lanzhou, 730000 Gansu China; 2 Clinical and Technical Support, Philips Healthcare, Xi’an, China; 3 Department of Radiology, Gansu Provincial Hospital, Lanzhou, 730000 Gansu China

**Keywords:** Prostate cancer, Magnetic resonance imaging, Radiomics, Aggressiveness, Gleason score, Positive needles

## Abstract

**Purpose:**

The Gleason score (GS) and positive needles are crucial aggressive indicators of prostate cancer (PCa). This study aimed to investigate the usefulness of magnetic resonance imaging (MRI) radiomics models in predicting GS and positive needles of systematic biopsy in PCa.

**Material and Methods:**

A total of 218 patients with pathologically proven PCa were retrospectively recruited from 2 centers. Small-field-of-view high-resolution T2-weighted imaging and post-contrast delayed sequences were selected to extract radiomics features. Then, analysis of variance and recursive feature elimination were applied to remove redundant features. Radiomics models for predicting GS and positive needles were constructed based on MRI and various classifiers, including support vector machine, linear discriminant analysis, logistic regression (LR), and LR using the least absolute shrinkage and selection operator. The models were evaluated with the area under the curve (AUC) of the receiver-operating characteristic.

**Results:**

The 11 features were chosen as the primary feature subset for the GS prediction, whereas the 5 features were chosen for positive needle prediction. LR was chosen as classifier to construct the radiomics models. For GS prediction, the AUC of the radiomics models was 0.811, 0.814, and 0.717 in the training, internal validation, and external validation sets, respectively. For positive needle prediction, the AUC was 0.806, 0.811, and 0.791 in the training, internal validation, and external validation sets, respectively.

**Conclusions:**

MRI radiomics models are suitable for predicting GS and positive needles of systematic biopsy in PCa. The models can be used to identify aggressive PCa using a noninvasive, repeatable, and accurate diagnostic method.

**Supplementary Information:**

The online version contains supplementary material available at 10.1007/s12672-024-00980-8.

## Introduction

Prostate cancer (PCa) has the highest incidence rate and the second-highest mortality rate in the male reproductive system worldwide [1]. Aggressive PCas have been proven to be a critical factor in the selection of clinical treatment strategies and assessment of prognosis. Patients with low-to-moderate aggressive PCas have satisfactory 5- and 10- year survival rates compared with those with highly aggressive PCas [2].

The Gleason score (GS) is the most widely used tool to assess the aggressiveness of PCa [3–5]. Studies have reported that the number of positive needles of systematic biopsy is also one of the indicators for assessing PCa aggressiveness [6]. A considerable difference in prognosis exists between patients with ≥ 50% positive needles and those with < 50% positive needles [7]. No study has yet reported to predict the number of positive needles.

Although magnetic resonance imaging (MRI) has a fair sensitivity for detecting and localizing PCa, certain small-volume lesions may be missed; thus, a relatively higher image quality and expertise of radiologists are required for accurate diagnosis [8, 9]. Radiomics is currently regarded as a promising noninvasive tool to characterize PCa aggressiveness. Previous studies [3, 10] have demonstrated that the machine learning model constructed by extracting MRI-based radiomics features from the whole prostate has satisfactory performance in predicting GS, and the area surrounding the tumor lesion provides information related to GS. Therefore, in this study, we aimed to develop radiomics models based on prostate gland segmentation to predict PCa aggressiveness regarding GS and positive needles of systematic biopsy.

## Materials and methods

### Patients

This study received ethics approval from the Medical Ethics Committee of Gansu Provincial Hospital (2022–458), and all patients were exempted from signing an informed consent form. Furthermore, all methods employed in this study were performed in strict accordance with the relevant guidelines and regulations around a local institution’s radiomics model.

The clinicopathologic data on patients with PCa were collected retrospectively from the electronic medical systems of 2 centers (Center A, Gansu Provincial Hospital from January 2018 to July 2023; Center B, Zhangye People's Hospital Affiliated to Hexi University from April 2020 to August 2023).

The clinical data, including age, serum total prostate-specific antigen (TPSA) level, serum-free prostate-specific antigen (FPSA) level, FPSA/TPSA, alkaline phosphatase (ALP) level, GS, and positive needle biopsy. GS and positive needle biopsy were obtained from medical records and pathologic reports. In this study, PSA density (PSAD) was calculated using the following formula: PSAD = TPSA/VPG, where VPG is the volume of the whole prostate gland. The value of VPG was obtained using small-field-of-view high-resolution T2-weighted imaging (sFOV HR-T2WI).

**Fig. 1:**
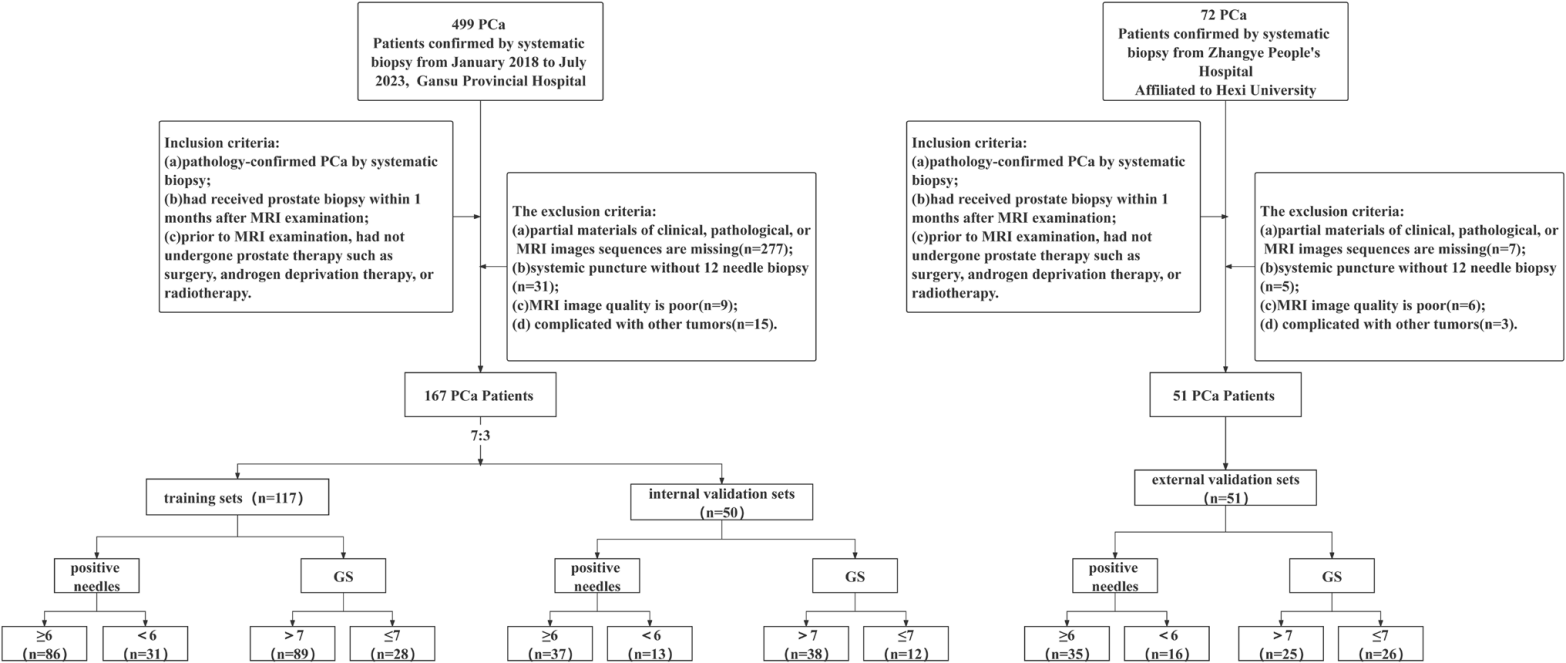
Flowchart of patient inclusion and exclusion criteria

The inclusion criteria were as follows: (a) pathologically confirmed PCa by systematic biopsy; (b) prostate biopsy performed within 1 month after MRI examination; and (c) no prostate therapy, such as surgery, androgen deprivation therapy, or radiotherapy, administered prior to MRI examination. The exclusion criteria were as follows: (a) clinical, pathologic, or MRI imaging data missing; (b) systemic puncture without 12-needle biopsy; (c) poor MRI image quality; and (d) presence of other tumors. The general flowchart of this study is depicted in Fig. 1.

### MRI acquisition

All enrolled patients underwent pelvic MRI examination on a 3.0-T MRI scanner (MAGNETOM Skyra, Siemens Healthcare, Erlangen, Germany; Siemens lumina 214,707) with the 18-channel phased-array abdominal coil. The center of the coil was aligned just above the pubic symphysis, and the coil was secured using an abdominal strap to minimize the influence of respiratory motion artifacts in the abdomen. The set image protocol included sagittal turbo spin-echo–T2WI and axial fat-suppressed–T2WI, axial diffusion weighted imaging (DWI) (*b* values of 50 and 1000) with apparent diffusion coefficient (ADC) maps, sFOV high-resolution turbo T2-weighted sequences on the axial planes, and post-contrast delayed scan sequences. The MRI data sets were retrieved from the picture achieving and communication system for further image processing. The MRI scan parameters are detailed in Table S1.

### The method of systematic biopsy for PCa

All patients were diagnosed of PCa by ultrasound guided transrectal prostate biopsy using the standard 10 + X puncture method (1 ~ 3 cores). The biopsy specimens were sent to the pathology department for routine histopathological examination, and the results were interpreted and recorded by a professional pathologist specializing in the urinary system.

### Region of interest segmentation

**Fig. 2:**
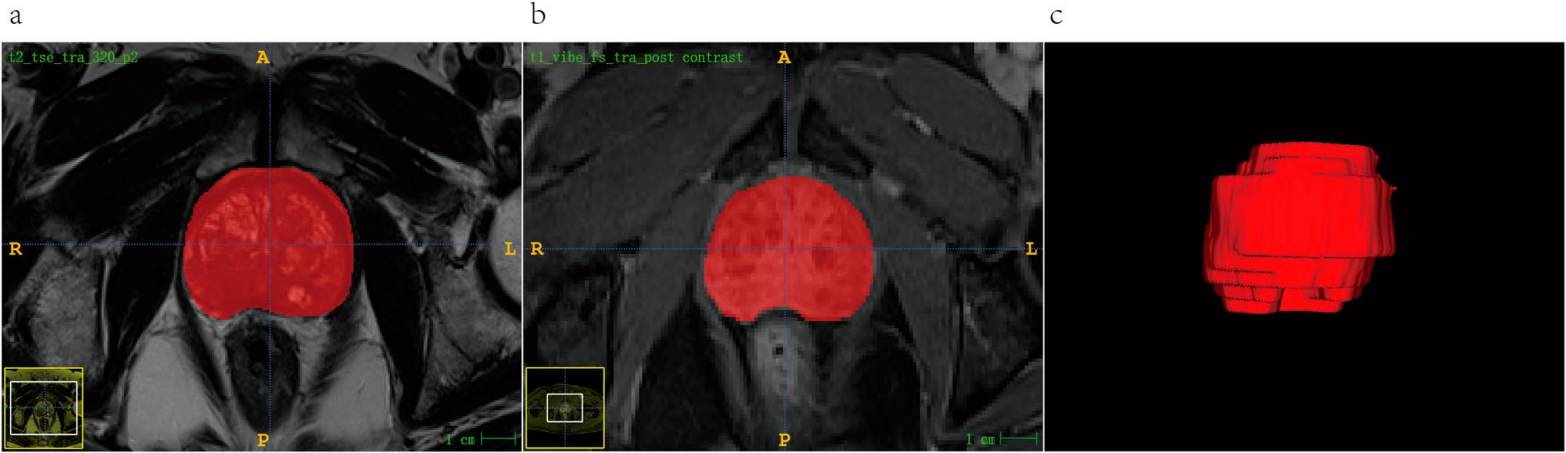
An example of the manual segmentation of prostate glands using sFOV HR-T2WI and post-contrast delayed scan sequences. **a** Gland segmentation on sFOV HR-T2WI; **b** gland segmentation on post-contrast delayed scan; and **c** 3-dimensional volume of interest

The sFOV HR-T2WI and post-contrast delayed scan images were imported into the open-source ITK-SNAP software (version 3.8.0, www.itksnap.org) in Digital Imaging and Communications in Medicine (DICOM) format. A radiologist with 5 years of experience in diagnosing prostate diseases routinely performed manual segmentation of the region of interest (ROI) layer by layer on the sFOV HR-T2WI images and post-contrast delayed scan images, avoiding the patient’s urethra, ejaculatory ducts, seminal vesicles, and the base of the seminal vesicles. Considering the necessity of overall ROI analysis in the heterogeneity assessment, the ROI included areas of hemorrhage, necrosis, cystic changes, and calcifications. After a rough outlining of the prostate gland, the software automatically generated the 3-dimensional (3D) volume of interest (VOI) for the entire prostate. Figure 2 depicts an example of the 3D manual segmentation. Figure S1 shows MRI images and pathological results of two PCa patients. A random sample of 30 patients was selected, and the same radiologist performed ROI after 3 months. This was conducted to assess the reproducibility and stability of radiomics features.

### Radiomics feature extraction

FeAture Explorer (FAE), which is a visualization program (version 0.5.5, https://github.com/salan668/FAE), was used for feature extraction after completing segmentation. A linear interpolation was adopted to resample the voxel size of the image to an isovolumetric voxel (1 × 1 × 1 mm^3^) before feature extraction. The voxel intensities of the image discretization were applied with a fixed bin width of 25 to reduce the influence of various machine types and scanning parameters on radiomics features. A series of quantitative radiomics features were extracted from the VOIs of axial sFOV HR-T2WI and post-contrast delayed scan images. The radiomics features were of 7 types: (a) first order; (b) shape-based; (c) gray-level co-occurrence matrix; (d) gray-level run-length matrix; (e) gray-level size zone matrix; (f) gray-level dependence matrix; and (g) neighboring gray-tone difference matrix.

### Feature selection and radiomics model establishment

For GS prediction, we performed upsampling of both positive and negative samples to reduce the imbalance in the classification training set data. The *Z*-score was used to normalize the features. For positive needle prediction, we performed SMOTE of both positive and negative samples to reduce the imbalance in the classification training set data. The Minimax was used to normalize the features. Furthermore, we applied Pearson correlation coefficients (PCC) on each pair of features to decrease the dimensionality of the feature matrix. We casually removed one of them with PCC > 0.9.

The analysis of variance (ANOVA), relief algorithm, recursive feature elimination, and Kruskal–Wallis test were used to select the most optimal features with nonzero coefficients from the retained standardized features and establish the radiomics signature. The support vector machine, linear discriminant analysis, logistic regression (LR), and LR using the least absolute shrinkage and selection operator were used to obtain the optimal feature combination based on accuracy step by step. We used the tenfold cross-validation to set the parameters according to the model performance on the validation data sets.

### Predictive performance of the radiomics models

The discrimination ability of the radiomics models was assessed and validated in the training, internal validation, and external validation sets using the area under the curve (AUC) of the receiver operating characteristic (ROC). Simultaneously, the accuracy, sensitivity, specificity, positive predictive value (PPV), and negative predictive value (NPV) were calculated based on the cutoff value that maximized the Youden index. The Matthews correlation coefficient (MCC) was a scalar measure commonly used to assess classification quality. The goodness-of-fit of the radiomics models was estimated using the calibration curve and the Hosmer–Lemeshow test. The decision curve analysis (DCA) demonstrated the clinical net benefits of the radiomics models [11].

### Statistical analysis

The data were statistically analyzed using the SPSS software (version 21.0, IBM, https://www.ibm.com/cn-zh/analytics/spss-statistics-software), GraphPad Prism (version 9.0.0, https://www.graphpad.com/), and R software (version 4.3.0). The Kolmogorov–Smirnov test was used to assess whether the continuous variable was normally distributed. Continuous variables conforming to a normal distribution were expressed as means ± standard deviation. The differences between 3 groups were evaluated using ANOVA and those between 2 groups using the independent-samples *t* test when appropriate. Qualitative and continuous variables with a nonnormal distribution were expressed as the medians (lower and upper quartiles). The differences among 3 groups were first tested using the Kruskal–Wallis test; if a considerable difference was identified, the difference between 2 groups was evaluated using the Mann–Whitney *U* test when appropriate. The Spearman rank correlation analysis was used to evaluate the correlation between positive needles and GS of systematic biopsy in PCa when the continuous variables were not normally distributed. A *P* value < 0.05 indicated a statistically significant difference.

## Results

### Clinical characteristics of patients

From Center A, 167 eligible patients were finally included in this study. They were randomly divided into training sets (*n* = 117) and internal validation sets (*n* = 50) at a ratio of 7:3 adopting the stratified sampling method. From Center B, 51 eligible patients were finally included in external validation sets (Fig. 1).

For GS prediction, the basic clinical information of patients in the training, internal validation, and external validation sets is presented in Table 1. Of the patients, 28 (23.9%), 12 (24%), and 26 (51%) in the training, internal validation, and external validation sets, respectively, had GS ≤ 7 (Fig. 1). For positive needle prediction, a comparison of the clinical features of the patients in the training, internal validation, and external validation sets is presented in Table 2. Of the patients, 31 (26.5%), 13 (26%), and 16 (11.8%) in the training, internal validation, and external validation sets, respectively, had positive needles < 6 (Fig. 1). Age, TPSA, FPSA, FPSA/TPSA, PSAD, and ALP were not statistically significantly different among the training, internal validation, and external validation sets (*P* > 0.05) (Table 2); however, TPSA, FPSA, FPSA/TPSA, and PSAD in the GS ≤ 7 and GS > 7 groups differed considerably (*P* < 0.05) (Table 3). Moreover, TPSA, FPSA, FPSA/TPSA, PSAD, and ALP in the positive needles ≥ 6 group were higher than those in the positive needles < 6 group, and the differences were statistically significant (*P* < 0.05) (Table 4).

**Table 1:** Clinical characteristics of patients for GS prediction

Variable	Training set	Internal validation set	External validation set	*P*
Age (year)	72.66 ± 7.19	72.14 ± 8.39	73.55 ± 6.83	0.622^a^
TPSA	46.81 (21.19, 100.00)	100.00 (31.23, 100.00)	45.24 (21.76, 100.00)	0.199^b^
FPSA	8.37 (2.85, 29.70)	13.03 (2.77, 30.00)	6.43 (2.93, 15.30)	0.441^b^
FPSA/TPSA	0.20 (0.12, 0.30)	0.21 (0.11, 0.30)	0.16 (0.11, 0.23)	0.306^b^
ALP	85.00 (67.00, 145.19)	90.50 (69.75, 138.30)	80.00 (60.00, 124.00)	0.143^b^
PSAD	1.14 (0.54, 1.87)	1.22 (0.445, 1.97)	1.15 (0.72, 2.12)	0.649^b^

^a^Statistical analysis performed using analysis of variance

^b^Statistical analysis performed using the Kruskal–Wallis test

*ALP* Alkaline phosphatase, *FPSA* free prostate-specific antigen, *GS* Gleason score; PSAD; TPSA, total prostate-specific antigen

**Table 2:** Clinical characteristics of patients for positive needles

Variable	Training set	Internal validation set	External validation set	*P*
Age (year)	72.73 ± 7.34	71.98 ± 8.07	73.55 ± 6.83	0.567^a^
TPSA	68.49 (24.67, 100.00)	49.72 (21.31, 100.00)	45.24 (21.76, 100.00)	0.551^b^
FPSA	8.93 (2.93, 30.00)	8.75 (2.21, 27.06)	6.43 (2.93, 15.30)	0.479^b^
FPSA/TPSA	0.20 (0.11, 0.30)	0.17 (0.12, 0.30)	0.16 (0.11, 0.23)	0.308^b^
ALP	93.00 (70.00, 145.19)	78.14 (64.00, 145.19)	80.00 (60.00, 124.00)	0.104^b^
PSAD	1.16 (0.58, 1.84)	1.11 (0.41, 1.96)	1.15 (0.72, 2.12)	0.623^b^

^a^Statistical analysis performed using analysis of variance

^b^Statistical analysis performed using the Kruskal–Wallis test

*ALP* Alkaline phosphatase, *FPSA* free prostate-specific antigen, *PSAD* TPSA, total prostate-specific antigen

**Table 3:** Clinical characteristics of patients with GS ≤ 7 and GS > 7

Variable	Total (*n* = 218)	GS > 7 (*n* = 152)	GS ≤ 7 (*n* = 66)	*Z*/*t*	*P*
Age (year)	72.5 ± 7.384	72.80 ± 7.579	72.62 ± 6.968	− 0.172	0.864^a^
TPSA	59.42 (21.84, 100.00)	76.42 (34.75, 100.00)	30.50 (12.34, 75.11)	− 4.546	<0 .001^b^
FPSA	8.44 (2.87, 29.69)	12.41 (4.05, 30.00)	3.35 (1.56, 9.99)	− 4.867	<0 .001^b^
FPSA/TPSA	0.19 (0.11, 0.30)	0.22 (0.12, 0.30)	0.14 (0.11, 0.21)	− 3.362	0.001^b^
ALP	84.00 (66.26, 144.25)	85.00 (68.02, 145.19)	81.00 (63.00, 114.75)	− 1.696	0.090^b^
PSAD	1.15 (0.57, 1.88)	1.33 (0.72, 1.98)	0.72 (0.35, 1.58)	− 2.995	0.03^b^

^a^Statistical analysis performed using the independent-samples *t* test

^b^Statistical analysis performed using the Mann–Whitney *U* test

*ALP* Alkaline phosphatase, *FPSA* free prostate-specific antigen, *GS* Gleason score, *PSAD* TPSA, total prostate-specific antigen

**Table 4:** Clinical characteristics of patients with positive needles ≥ 6 and < 6

Variable	Total (*n* = 218)	Positive score ≥ 6 (*n* = 158)	Positive score < 6 (*n* = 60)	*Z*/*t*	*P*
Age (year)	72.5 ± 7.384	72.56 ± 7.589	73.25 ± 6.849	0.647	0.519^a^
TPSA	59.42 (21.84, 100.00)	82.61 (39.17, 100.00)	19.70 (11.15, 52.05)	− 6.559	< 0.001^b^
FPSA	8.44 (2.87, 29.69)	13.07 (4.49, 30.00)	2.48 (1.47, 6.23)	− 6.714	< 0.001^b^
FPSA/TPSA	0.19 (0.11, 0.30)	0.22 (0.12, 0.30)	0.13 (0.10, 0.19)	− 4.165	<0 .001^b^
ALP	84.00 (66.26, 144.25)	85.50 (68.05, 145.19)	76.00 (63.50, 123.00)	− 2.126	0.034^b^
PSAD	1.15 (0.57, 1.88)	1.44 (0.81, 2.18)	0.54 (0.29, 1.12)	− 5.910	< 0.001^b^

^a^Statistical analysis performed using the independent-samples *t* test

^b^Statistical analysis performed using the Mann–Whitney *U* test

*ALP* Alkaline phosphatase, *FPSA* free prostate-specific antigen, *PSAD*; TPSA, total prostate-specific antigen

### Radiomics feature extraction and feature selection

**Fig. 3:**
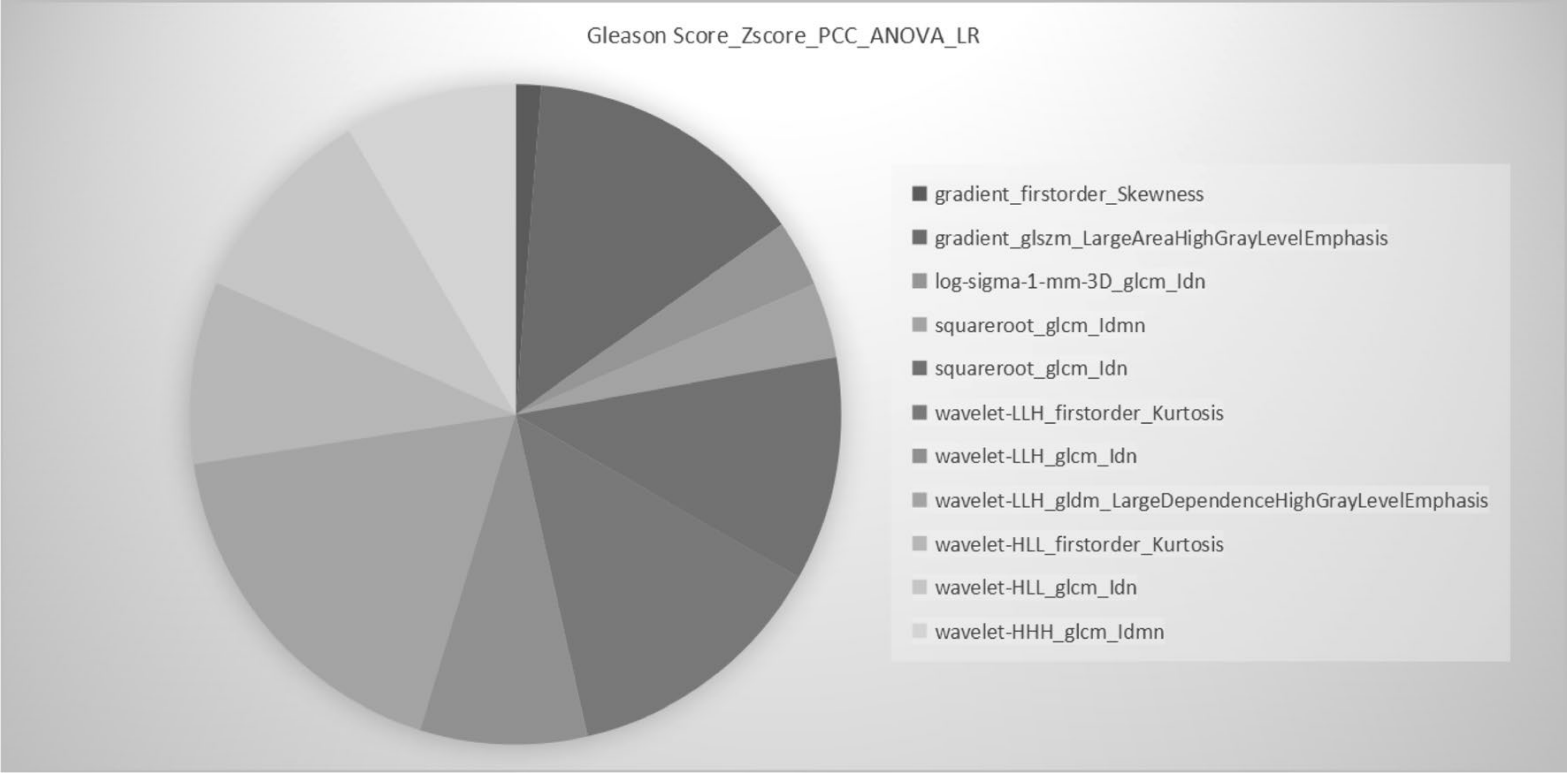
Optimal radiomics model (Zscore_PCC_ANOVA_LR) for GS prediction. Different colors represent different radiomics features, and the area represents the weighted contribution of each radiomics feature in the radiomics model. The feature with the highest weight for GS prediction was wavelet-LLH_gldm_LargeDependenceHighGrayLevelEmphasis. Zscore is the method of normalizing the features for GS prediction. PCC is a feature dimension reduction method for GS prediction. ANOVA is a feature selection method for GS prediction. LR is the classifier for radiomics model for GS prediction. *PCC * Pearson Correlation Coefficients. *ANOVA * analysis of variance. *LR*  logistic regression

**Fig. 4:**
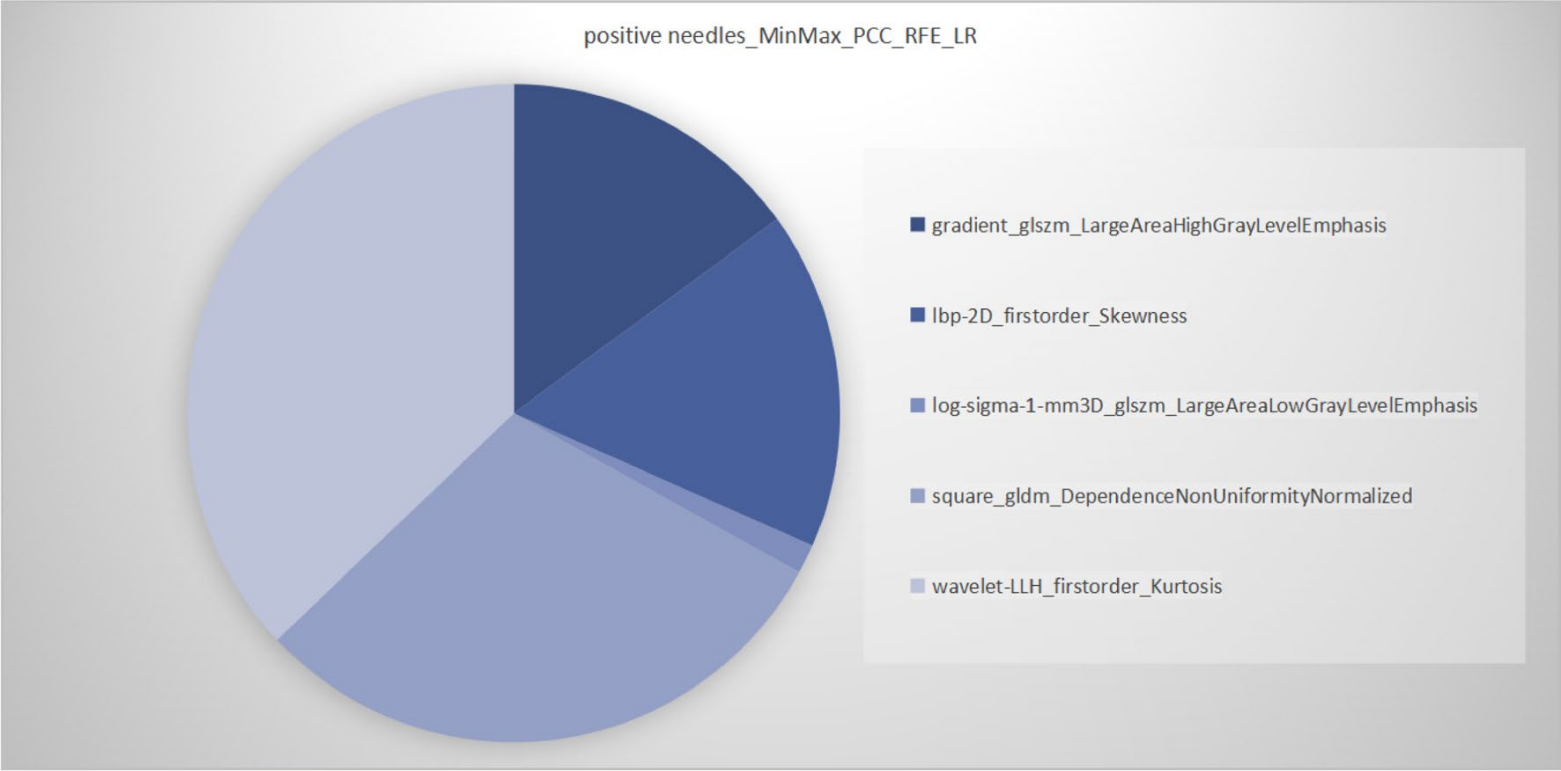
Optimal radiomics model (MinMax_PCC_RFE_LR) for positive needle prediction. Different colors represent different radiomics features, and the area represents the weighted contribution of each radiomics feature in the radiomics model. The feature with the highest weight for GS prediction was wavelet-LLH_firstorder_Kurtosis. MinMax is the method of normalizing the features for positive needle prediction. PCC is a feature dimension reduction method for positive needle prediction. RFE is a feature selection method for positive needle prediction. LR is the classifier for radiomics model for positive needle prediction. *PCC* Pearson correlation coefficients. *RFE*  Recursive feature elimination. LR  logistic regression

The reliability of the extracted radiomics features in terms of Intraclass correlation coefficient (ICC) for all features was quantified, with mean ICC values of 0.982. The top 11 features were chosen as the prime feature subset for the GS prediction (Table S2 and Fig. 3), whereas the top 5 features were chosen for positive needle prediction (Table S3 and Fig. 4).

### GS and positive needle prediction models

**Fig. 5:**
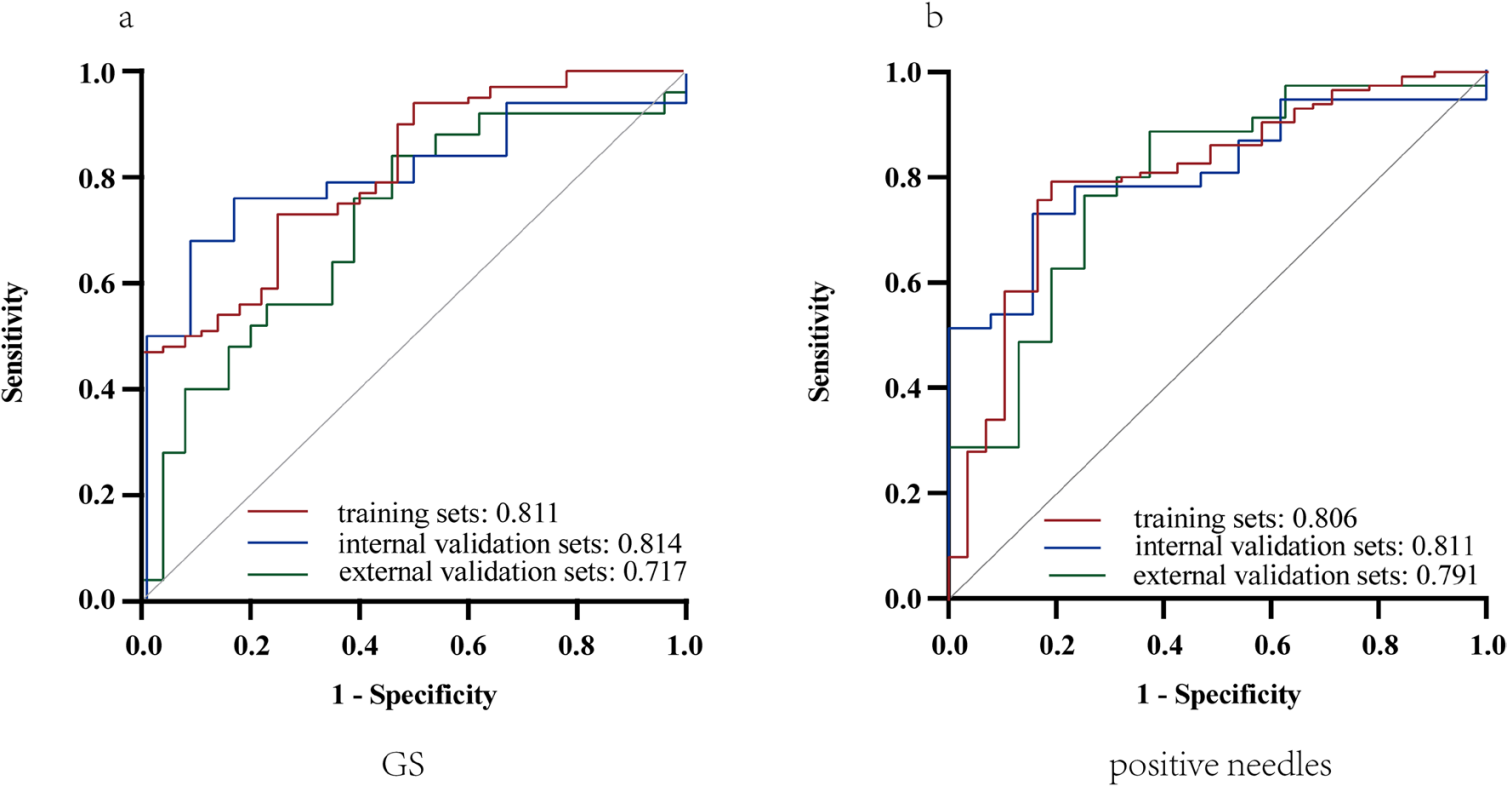
Performance of the radiomics models for GS **a** and positive needle **b** prediction in the training, internal validation, and external validation sets

The GS and positive needles exhibited a moderate positive correlation (*r* = 0.415, *P* < 0.01). The results demonstrated that the leading model for predicting GS and positive needles was LR. For GS prediction, the AUC of the radiomics models was 0.811, 0.814, and 0.717 in the training, internal validation, and external validation sets, respectively (Table S4 and Fig. 5a). The radiomics models had an accuracy of 74%, 74%, and 69%; a sensitivity of 75%, 92%, and 54%; a specificity of 73%, 68%, and 84%; a PPV of 47%, 48%, and 78%; an NPV of 90%, 96%, and 64%; and an MCC of 42%, 51%, and 40% in the training, internal validation, and external validation sets, respectively (Table S4).

For positive needle prediction models, the AUC was 0.806, 0.811, and 0.791 in the training, internal validation, and external validation sets, respectively (Table S5; Fig. 5b). The radiomics models had an accuracy of 79%, 76%, and 76%; a sensitivity of 81%, 85%, and 75%; a specificity of 79%, 73%, and 77%; a PPV of 58%, 52%, and 60%; an NPV of 92%, 93%, and 87%; and an MCC of 55%, 51%, and 50% in the training, internal validation, and external validation sets, respectively (Table S5).

### Performance assessment of the radiomics models

The calibration curve and Hosmer–Lemeshow test statistic exhibited favorable calibration in the training sets, which was confirmed in the internal and external validation sets (Fig. S2, S3a). Subsequently, the DCA was performed to analyze the clinical practicability of the radiomics models. The patients would benefit more from the radiomics models than either the treatment of all schemes or the no-treatment regimen (Fig. S2, S3b). Furthermore, the prediction efficacy was compared by ROC analyses in the training, internal validation, and external validation sets (Fig. 5).

## Discussion

Currently, tissue biopsy remains the gold standard for PCa diagnosis. Pathologic biopsy is also a common method for estimating PCa aggressiveness, but it has various complications limiting its clinical use. Furthermore, this evaluation typically relies on a solitary biopsy of a potentially heterogeneous tumor, which can merely capture a snapshot of its biological characteristics [12]. In the present dual-center retrospective study, we built and tested radiomics models for predicting PCa aggressiveness regarding GS and positive needles of systematic biopsy. The models were validated using internal and external validation sets. The established radiomics models provided a quantifiable and individualized tool for predicting PCa aggressiveness, thus helping in clinical decision-making.

Serum PSA is a specific marker of PCa and the only tumor marker with organ specificity. In this study, TPSA, FPSA, FPSA/TPSA, and PSAD in the GS ≤ 7 and GS > 7 groups differed considerably. It may be due to the biological differences between tumors with different grades. With the increase of serum PSA related indicators, GS will also increase. Moreover, TPSA, FPSA, FPSA/TPSA, and PSAD in the positive needles ≥ 6 group were higher than those in the positive needles < 6 group. We guessed that the number of positive needles is an indicator of the extent and localization of cancerous tissue of PCa. A higher number of positive needles (≥ 6) suggests a more extensive disease involvement.

Patient management for PCa requires an accurate evaluation of potential tumor aggressiveness [13, 14]. GS is a histopathological grading system used to assess the aggressiveness of PCa. Tumors with a higher GS are typically more aggressive and have a poorer prognosis. Previous studies investigated the radiomics methods to identify GS for estimating PCa aggressiveness. Gong’s group [15] presented a biparametric MRI radiomics for discriminating between patients with GS ≤ 7 and those with GS > 7, achieving satisfactory performance, with AUCs of 0.811 and 0.788 in the training and test cohorts, respectively. In another study, a multiparametric MRI-based radiomics signature demonstrated the potential to noninvasively distinguish between indolent and aggressive PCa [16]. However, a few studies mainly predicted GS, paying little attention to positive needles. Besides, they were single-center studies without external validation, whose results might be less robust and generalizable. The present study was a 2-center clinical study. We predicted simultaneously GS and positive needles of systematic biopsy based on whole prostate gland segmentation. This method may aid clinical doctors in evaluating comprehensively PCa aggressiveness, thus enabling personalized medicine. Highly aggressive PCa progresses rapidly and requires early intervention such as radical surgery or radiation therapy.

This study employed a radiomics analysis based on the whole prostate gland. It developed a noninvasive method for predicting GS ≤ 7 and GS > 7, and positive needles ≥ 6 and positive needles < 6, among patients with PCa. In agreement with previous studies [17, 18], LR was chosen as the classifier to construct radiomics models in this study, suggesting its advantages in assessing PCa aggressiveness. One of the reasons might be that LR was particularly effective in binary classification tasks [19]. For GS prediction, the radiomics models demonstrated a moderate-to-good diagnostic performance with an AUC of 0.811 [95% confidence interval (CI), 0.73–0.90] in the training sets, 0.814 (95% CI 0.69–0.93) in the internal validation sets, and 0.717 (95% CI 0.57–0.86) in the external validation sets. For positive needle prediction, the radiomics models demonstrated satisfactory predictive efficiency with an AUC of 0.806 (95% CI 0.71–0.89) in the training sets, 0.811 (95% CI 0.69–0.93) in the internal validation sets, and 0.791 (95% CI 0.65–0.93) in the external validation sets. Considering the external validation sets, the radiomics models performed satisfactorily in identifying positive needles compared with identifying GS. The reason might be that positive needles included more lesions and invaded regions with PCa. Specifically, 11 features were chosen for the GS prediction, whereas 5 features were chosen for positive needle prediction. Among all the features, the texture and wavelet features were vital, providing more information regarding tumor heterogeneity, which has been confirmed in several other studies [20–22]. We further analyzed the radiomics features and observed that Gradient_glszm_LargeAreaHighGrayLevelEmphasis and wavelet-LLH_firstorder_Kurtosis were common features of GS and positive needle prediction, although they did not contribute the most. The texture features can serve as a biomarker for predicting the presence of clinically remarkable PCa [23]. The wavelet filter disassembles the original images in various directions and reveals multidimensional spatial heterogeneity, which can assist in revealing tumor heterogeneity that may not be detectable in the original images [24, 25]. Therefore, we concluded that the texture and wavelet features might be the most helpful in predicting GS and positive needles of systematic biopsy to estimate PCa aggressiveness. Clinicians might be alerted to a potentially highly aggressive PCa using radiomics as a noninvasive method in our workflow.

Numerous studies have suggested the wide use of MRI for the diagnosis, staging, and treatment monitoring of various tumor types [26–28]. In this study, we initially predicted the PCa aggressiveness from sFOV HR-T2WI and post-contrast delayed scan sequences. The use of only these two sequences may limit the diversity of radiomic features that can be extracted. Different imaging modalities and sequences capture different aspects of tissue properties, and a more comprehensive approach incorporating multiple sequences and modalities could provide a richer set of features for analysis. However, the features that established radiomics models were all derived from the VOI on sFOV HR-T2WI series, indicating the crucial role of sFOV HR-T2WI images in providing aggressiveness-relevant information. The heavily weighted radiomic features from sFOV HR-T2WI series may effectively reflect more potential morphological and heterogeneity features of tumors, with higher spatial resolution and contrast. The result of this study was in line with that of previous studies [29, 30]. T2WI was considered more valuable than contrast-enhanced scanning sequences in reflecting tumor heterogeneity. Hence, choosing a valuable sequence is vital, avoiding time-consuming and laborious image segmentation. Therefore, we concluded that radiomics based on sFOV HR-T2WI might contribute to assessing PCa aggressiveness and risk stratification without additional MRI sequences such as dynamic contrast-enhanced MRI.

A large number of studies confirmed that tumor heterogeneity was not only solely determined by the tumor itself but also closely related to TME, which was perceived as a major determinant of cancer progression and aggressiveness [31, 32]. In this study, the prostate gland segmentation included lesion and TME information, providing a more comprehensive description of tumor-related information, which was helpful for tumor diagnosis and prognosis assessment. Furthermore, according to previous studies, approximately 50% of the radiomics features for prostate lesion segmentation were unstable, whereas only 20% of the radiomics features for gland segmentation were unstable [3]. In this study, only 1% of radiomics features from the gland had an ICC value < 0.8, indicating that the radiomics features from the whole prostate exhibited improved reproducibility and stability. This also highlighted the possibility of achieving a fully automatic segmentation of the whole prostate gland and promoting the noninvasive prediction of PCa aggressiveness in the future [33].

Data balancing tools, such as upsampling, downsampling, or SMOTE, have been demonstrated to considerably improve the predictive performance of radiomics models. In this study, the performance of radiomics models distinctly improved after data upsampling or SMOTE, which was in an excellent agreement with previous findings [3, 34].

This study had several limitations. First, it was a retrospective study, and although external validation data was included, the total sample size collected was small. In future studies, we plan to increase the sample size and conduct more external validations from multiple centers to obtain higher levels of clinical evidence. Second, only sFOV HR-T2WI and post-contrast delayed scan sequences were used. Other sequences, such as DWI, ADC, and dynamic contrast-enhanced MRI, could be worth exploring. Third, the radiomics models based on lesion segmentation was not involved, which will be investigated in our future works. Furthermore, the pathologic evaluations of 2 hospitals in this study did not guarantee that the results were obtained by the same urology specialist pathologist at the same time, introducing a certain degree of subjective variability.

## Conclusions

In conclusion, the results of this dual-center study demonstrated that the radiomics models based on the prostate gland achieved improved performance for predicting PCa aggressiveness. These findings may potentially assist in identifying preoperative high-risk patients with PCa and developing individualized treatment, unnecessary needles biopsy was avoided and the life quality of patients was ameliorated.

### Supplementary Information

Additional file1 (CSV 265 KB)

Additional information, Intraclass correlation coefficient (ICC) results of all radiomics features.

Additional file2 (DOCX 35598 KB)

Additional Tables and Figures, Tables **S1**-**S6**, Figures **S1**-**S3**.

## Data Availability

The data that support the findings of this study are available on request from the corresponding author.
