# Supplementary material for: Prediction of prostate cancer aggressiveness using magnetic resonance imaging radiomics: a dual-center study
Source: Discov Oncol. 2024 Apr 16;15:122. doi: 10.1007/s12672-024-00980-8 (PMC11019191; doi:10.1007/s12672-024-00980-8)
Supplement: Supplementary file 2 — Additional file2 (DOCX 35598 KB) [file 12672_2024_980_MOESM2_ESM.docx]

**Prediction of Prostate Cancer Aggressiveness Using Magnetic Resonance Imaging Radiomics: A Dual-center Study**

Nini Pan^1^, Liuyan Shi^1^, Diliang He^1^, Jianxin Zhao^1^, Lianqiu Xiong^1^, Lili Ma^1^, Jing Li^1^, Kai Ai^2^, Lianping Zhao^3^, Gang Huang^3^*

^1^ The First Clinical Medical College of Gansu University of Chinese Medicine, Lanzhou, Gansu 730000, China

^2^ Clinical and Technical Support, Philips Healthcare, Xi’an, China

^3^ Department of Radiology, Gansu Provincial Hospital, Lanzhou, Gansu 730000, China

*Corresponding author, E-mail: keen0999@163.com

**Table S1** MRI parameter protocol from Centers A and B

| Sequence parameter | Gansu Provincial Hospital | | Zhangye People's Hospital Affiliated to Hexi University | |
| --- | --- | --- | --- | --- |
|  | sFOV HR-T2WI | Post-contrast delayed | sFOV HR-T2WI | Post-contrast delayed |
| TR (ms) | 7500 | 4.21 | 4000 | 5.5 |
| TE (ms) | 101 | 1.98 | 134 | 2.46 |
| Thickness (mm) | 3 | 2 | 3 | 3 |
| Matrix | 320 × 320 | 320 × 240 | 340 × 340 | 109 × 109 |
| FOV (mm) | 180 × 100 | 320 × 100 | 220 × 100 | 350 × 100 |
| Flip angle (degree) | 160 | 9 | 160 | 9 |

**Table S2** The 11 features for GS prediction

| Feature | Coefficient |
| --- | --- |
| gradient_firstorder_Skewness | −0.085 |
| gradient_glszm_LargeAreaHighGrayLevelEmphasis | −0.942 |
| log-sigma-1-mm-3D_glcm_Idn | 0.221 |
| squareroot_glcm_Idmn | 0.249 |
| squareroot_glcm_Idn | −0.738 |
| wavelet-LLH_firstorder_Kurtosis | −0.891 |
| wavelet-LLH_glcm_Idn | 0.558 |
| wavelet-LLH_gldm_LargeDependenceHighGrayLevelEmphasis | −1.202 |
| wavelet-HLL_firstorder_Kurtosis | −0.601 |
| wavelet-HLL_glcm_Idn | 0.669 |
| wavelet-HHH_glcm_Idmn | −0.575 |

**Table S3** The 5 features for positive needle prediction

| Feature | Coefficient |
| --- | --- |
| gradient_glszm_LargeAreaHighGrayLevelEmphasis | −2.525 |
| lbp-2D_firstorder_Skewness | 2.799 |
| log-sigma-1-mm3D_glszm_LargeAreaLowGrayLevelEmphasis | −0.245 |
| square_gldm_DependenceNonUniformityNormalized | −5.037 |
| wavelet-LLH_firstorder_Kurtosis | −6.252 |

**Table S4** Performance of the radiomics models for GS prediction in the training, internal validation, and external validation sets

|  | AUC (95% CI) | Accuracy | Youden index | Sensitivity | Specificity | PPV | NPV | MCC |
| --- | --- | --- | --- | --- | --- | --- | --- | --- |
| Training sets | 0.811 (0.726-0.897) | 0.735 | 0.480 | 0.75 | 0.730 | 0.467 | 0.903 | 0.421 |
| Internal validation sets | 0.814 (0.694-0.934) | 0.740 | 0.601 | 0.917 | 0.684 | 0.478 | 0.963 | 0.515 |
| External validation sets | 0.717 (0.572-0.862) | 0.686 | 0.379 | 0.539 | 0.840 | 0.778 | 0.636 | 0.396 |

AUC, Area under the curve; CI, confidence interval; GS, Gleason score; MCC, Matthews correlation coefficient; NPV, negative prediction value; PPV, positive prediction value

**Table S5** Performance of the radiomics models for positive needle prediction in the training, internal validation, and external validation sets

|  | AUC (95% CI) | Accuracy | Youden index | Sensitivity | Specificity | PPV | NPV | MCC |
| --- | --- | --- | --- | --- | --- | --- | --- | --- |
| Training sets | 0.806 (0.714-0.898) | 0.795 | 0.597 | 0.807 | 0.791 | 0.581 | 0.919 | 0.547 |
| Internal validation sets | 0.811 (0.689-0.933) | 0.760 | 0.576 | 0.846 | 0.730 | 0.524 | 0.931 | 0.512 |
| External validation sets | 0.791 (0.652-0.930) | 0.765 | 0.521 | 0.750 | 0.771 | 0.600 | 0.871 | 0.496 |

AUC, Area under the curve; CI, confidence interval; MCC, Matthews correlation coefficient; NPV, negative prediction value; PPV, positive prediction value

**Table S6** Clinical characteristics of PCa

| Variable |  |
| --- | --- |
| Age (year) |  |
| < 65 | 20/167 |
| ≥ 65 | 147/167 |
| PSA (ng/ml) |  |
| 0-10 | 14/167 |
| ＞10 | 153/167 |
| GS |  |
| 6 | 9/167 |
| 7 | 31/167 |
| 8 | 57/167 |
| 9 | 55/167 |
| 10 | 15/167 |
| positive needles |  |
| 1 | 6/167 |
| 2 | 10/167 |
| 3 | 13/167 |
| 4 | 11/167 |
| 5 | 5/167 |
| 6 | 10/167 |
| 7 | 4/167 |
| 8 | 11/167 |
| 9 | 9/167 |
| 10 | 16/167 |
| 11 | 15/167 |
| 12 | 57/167 |

**Fig. S1** Prostate cancer patient's MRI image and pathological results illustrated

a-c: a 82-year-old PCa patient, Gleason score = 7(4+3), posotive needles = 1; d-f: a 74-year-old PCa patient, Gleason score = 9(5+4), posotive needles = 9; a, d: sFOV HR-T2WI scan sequences; b, e: post-contrast delayed scan sequences; c, f: Loupe image of hematoxylin–eosin stain


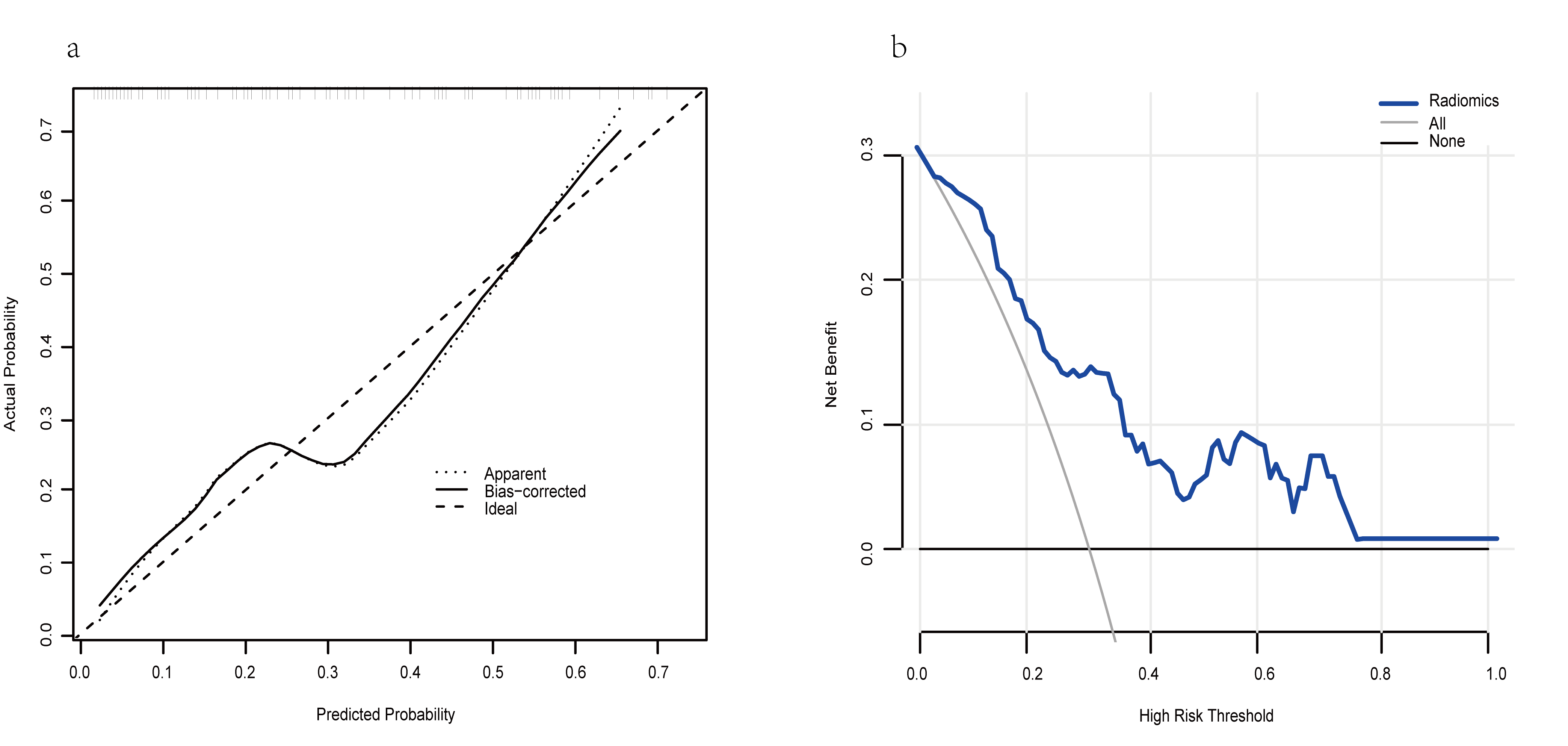


**Fig. S2** Calibration curve of radiomics models in the internal and external validation cohorts (a); and decision curve analysis of radiomics models (b) for GS prediction


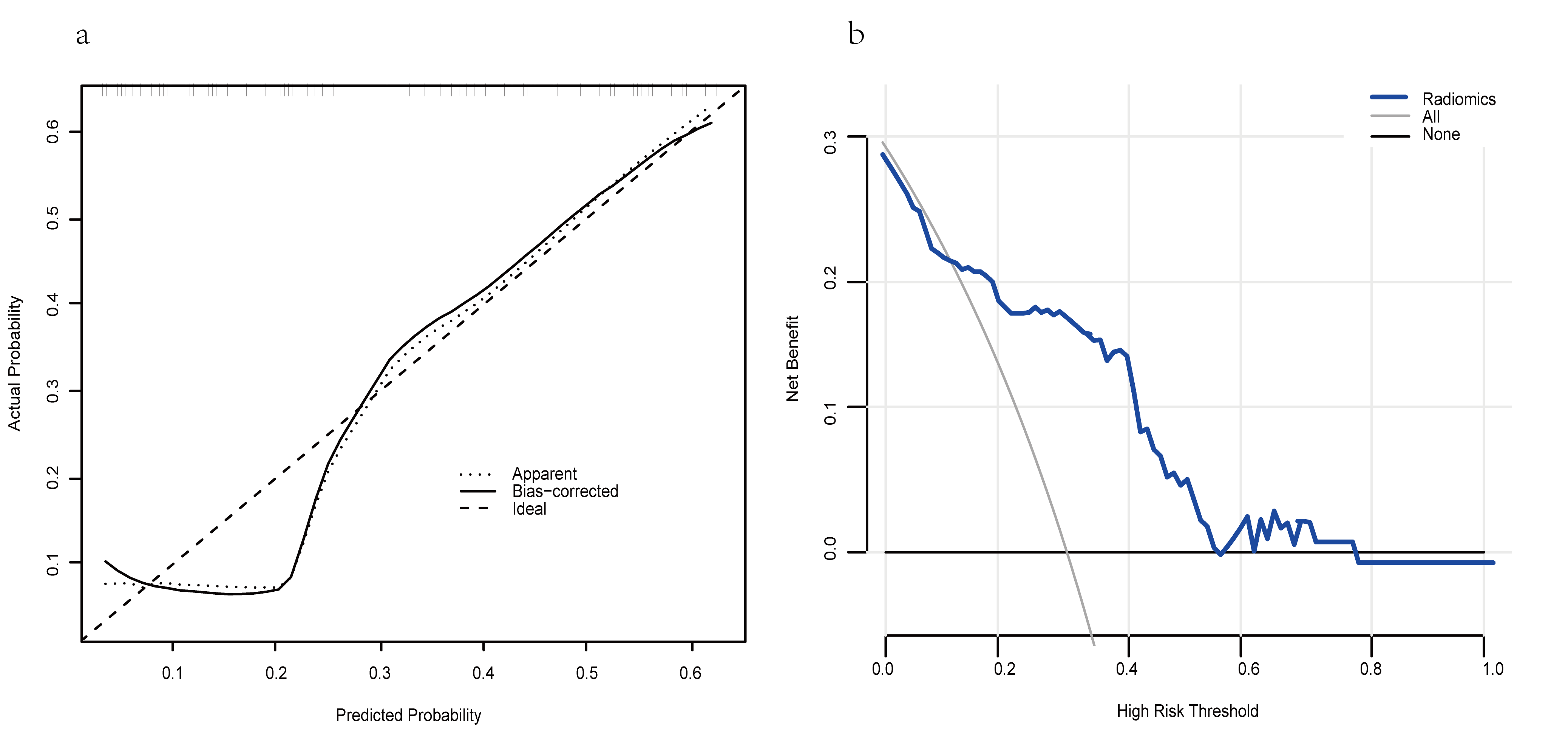


**Fig. S3** Calibration curve of radiomics models in the internal and external validation cohorts (a); and decision curve analysis of radiomics models (b) for positive needles prediction
